# Stimulus‐Induced Self‐Reinforcement in Supramolecular Bamboo Plastics toward Mechanical Robustness and Programmable Shapeability

**DOI:** 10.1002/advs.74426

**Published:** 2026-02-15

**Authors:** Jingcai Li, Geyuan Jiang, Suqing Zeng, Minxin Wang, Haipeng Yu, Dawei Zhao

**Affiliations:** ^1^ Key Laboratory On Resources Chemicals and Materials of Ministry of Education Shenyang University of Chemical Technology Shenyang P. R. China; ^2^ Key Laboratory of Bio‐based Material Science and Technology of Ministry of Education Northeast Forestry University Harbin P. R. China

**Keywords:** bioplastic, cellulose, mechanical robustness, self‐reinforcement, supramolecular network

## Abstract

The widespread use of petrochemical plastics has made environmental problems and health risks increasingly prominent. While bioplastics hold promise as alternatives, their limited heat resistance and shaping capabilities hinder widespread adoption in high‐performance engineering. Here, we present an innovative supramolecular network that utilizes cellulose as a molecular framework, complemented by acrylamide molecules for in situ polymerization. By applying ethanol for structural reconstruction, we create a self‐reinforcing bioplastic (S‐bioplastic) with a tensile strength of 76 MPa and a flexural modulus of 4.7 GPa. This S‐bioplastic supports multiple molding techniques—such as injection and compression molding—and exhibits remarkable environmental adaptability, with thermal stability up to 180°C and low‐temperature resilience to −196°C. Compared to conventional plastics, S‐bioplastic offers enhanced mechanical properties, biocompatibility, biodegradability, and recyclability, achieving a 95% retention of strength. A techno‐economic analysis underscores its value proposition. This research highlights a method for converting bamboo‐based materials into high‐value bioplastics, providing a promising strategy to address plastic pollution while developing lightweight, high‐performance materials for aerospace applications.

## Graphical abstract

A self‐reinforcement strategy is reported for the fabrication of bamboo plastics utilizing ethanol to induce the supramolecular network reconstruction of cellulose and polyacrylamide molecules. These bamboo plastics exhibit high mechanical strength, excellent thermal stability, resistance to low temperatures, and controllable shapeability. These characteristics position bamboo plastics as promising candidates for the development of lightweight, high‐performance materials for a range of applications.

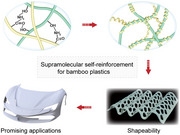

## 1 Introduction

**FIGURE 1:**
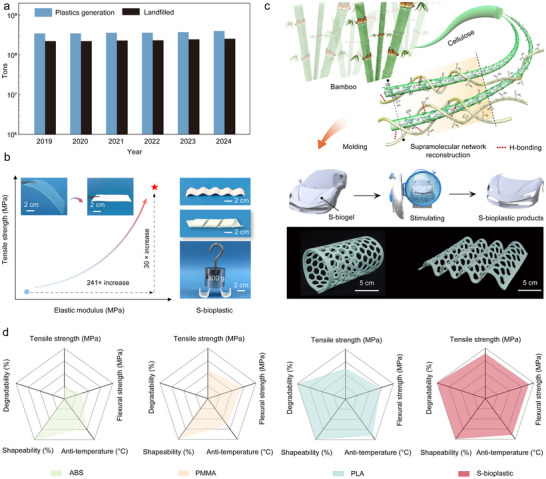
Supramolecular network structure design strategy and properties of S‐bioplastic. (a) Production and landfill volumes of petroleum‐based plastics based on statistics from 2019 to 2024, provided by Plastics Europe. (b) Comparison of tensile strength and elastic modulus of S‐biogel with S‐bioplastic. (c) Supramolecular network structure design strategy and molding process of S‐bioplastic. d) Radar plot to compare S‐bioplastic and commercial plastic (ABS, PMMA, and PLA).

Petroleum‐based plastics are widely used in medical, agricultural, industrial, and construction fields due to their lightweight, durable, and easy‐to‐mold properties [1, 2, 3, 4, 5]. With the development of the plastic industry and the widespread use and consumption of plastic products, nearly 400 million tons of plastic have been used annually over the past five years. However, only 10% of plastic waste is recycled (Figure 1a; Figure S1) [6, 7, 8, 9]. The remainder accumulates in landfills and gradually transforms into persistent microplastics that are difficult to decompose naturally, causing severe environmental pollution and health risks [10, 11, 12, 13, 14].

To achieve sustainable alternatives to plastics, in recent years, bioplastics developed from renewable green resources, such as polylactic acid (PLA), polyhydroxyalkanoates (PHA), and polyhydroxybutyrate (PHB), have been widely used in fields like food packaging and daily commodities [15, 16, 17, 18, 19, 20]. However, compared with traditional plastics, these bioplastics still have gaps in processability, heat resistance, and mechanical strength. This severely limits their application in high‐performance areas [21, 22, 23, 24]. Therefore, how to use green, renewable biomaterials to develop lightweight, high‐strength, and shapeable bioplastics that can replace non‐renewable traditional plastics remains a pressing scientific gap [25, 26, 27].

Bamboo, as a natural renewable resource, offers significant sustainability advantages, including a short growth cycle (harvestable in just 3–5 years), abundant reserves, and low cost [28, 29]. With properties like biodegradability, low density, and high mechanical strength, it is considered one of the most attractive raw materials for replacing petroleum‐based plastics [30, 31]. As an important component of bamboo, bamboo fibers exhibit unique multi‐scale structural characteristics—from macroscopic fiber bundles to microscopic fiber cells and nanoscale cellulose microfibrils, assembled layer by layer into a highly ordered reinforcement system [32, 33, 34, 35, 36]. While this structural feature gives bamboo fibers significantly higher strength and modulus than other plant fibers [37, 38], it inevitably causes problems like low flexibility and poor plasticity [39, 40]. Therefore, how to effectively restructure these fibers and achieve performance enhancement while maintaining original stiffness remains a challenge for the large‐scale application of bamboo‐based bioplastics.

Here, we propose a solvent‐induced strategy for the design of a self‐reinforcing supramolecular network. Bamboo cellulose is selected as the skeletal framework, and acrylamide molecules are introduced for in situ polymerization. When stimulated by ethanol, polyacrylamide (PAM) molecules curl and envelop the bamboo cellulose molecular chains, leading to the formation of a self‐reinforcing supramolecular configuration within S‐bioplastic. This S‐bioplastic demonstrates superior mechanical properties, surpassing those of most commercially available bioplastics, including PLA, PHA, and PHB. Additionally, S‐bioplastic demonstrates exceptional environmental tolerance with thermal stability up to 180°C and low‐temperature resistance down to ‐196°C. While maintaining performance advantages, S‐bioplastic also possesses good biocompatibility, natural biodegradability, and recyclability with 95% strength retention. These characteristics make S‐bioplastic a promising, sustainable, and scalable lightweight plastic, providing a viable solution for promoting industrialization and high‐performance development of bamboo‐based plastics, mitigating plastic pollution, and reducing fossil resource dependence.

## 2 Results and Discussion

### 2.1 Design Strategy and Properties of S‐bioplastic

We use the ionic liquid 1‐butyl‐3‐methylimidazolium chloride ([Bmim]Cl) to break the H‐bond network between bamboo fiber molecules [41, 42], forming a uniform bamboo cellulose/[Bmim]Cl system. Then, through solvent replacement, [Bmim]Cl is replaced with water to obtain a flexible bamboo cellulose hydrogel. Using the bamboo cellulose molecular chains as the framework, acrylamide molecules were introduced for in situ polymerization to prepare bamboo cellulose/polyacrylamide hydrogel (S‐biogel). Subsequently, S‐biogel was stimulated with ethanol. Under the stimulating effect of ethanol, the H‐bond network of S‐biogel begins to reconstruct. The cellulose molecular chains arrange more closely, and the H‐bond network is strengthened. At the same time, polyacrylamide molecules (PAM) form new H‐bonds with cellulose molecular chains through their ─CO─NH_2_ groups and wrap around the cellulose molecular chains, thereby preparing bamboo cellulose bioplastics (S‐bioplastic) with a more compact and coherent supramolecular network structure (Figure S2). The tensile strength of S‐biogel is 2.54 MPa, and the elastic modulus is 10.83 MPa. In contrast, the tensile strength and elastic modulus of S‐bioplastic increased by 30 times and 241 times, respectively (Figure 1b).

Benefiting from the self‐reinforcement behavior, S‐bioplastic can be easily twisted and shaped (Figure 1c). To verify the practical application of this plasticity, we wrapped the S‐biogel on a 3D mold of the target shape, and then transferred it to an ethanol solution container for processing, and the consolidated S‐bioplastic product was obtained. Based on this processing method, S‐bioplastic can be formed into actual products by simple coating, including S‐bioplastic products of a cylindrical honeycomb plate and a corrugated honeycomb plate (Figure 1c). This highlights the potential application value of S‐bioplastic in complex and precision devices.

To further address the issue of low toughness in bamboo‐based bioplastics, we studied the mechanical tensile properties of S‐bioplastic prepared with different concentrations of bamboo cellulose. The S‐bioplastic with a cellulose content of 57.37 ± 0.5 wt%, prepared by 15 wt% bamboo cellulose concentration, showed a significant tensile strength of 72 ± 3.3 MPa (Figure S3a). Moreover, a 0.68 g sample of S‐bioplastic can easily extract a weight of 5 kg, exceeding its own weight by more than 7000 times without fracturing (Figure S3b). This discovery highlights the great application prospects of S‐bioplastics in the fields of construction and engineering manufacturing. Then, compared with polyacrylamide (PAM) and lignocellulose/polyacrylamide (Cel/PAM), the S‐bioplastic exhibits good flexibility as well as significant strength and modulus (Figure S4), which fully demonstrates that bamboo fiber is an ideal choice for preparing high‐performance bioplastic materials.

We further summarized a comprehensive comparison between this bamboo cellulose bioplastic and some representative commercial plastics (Figure 1d), such as acrylonitrile‐butadiene‐styrene copolymer (ABS), polymethyl methacrylate (PMMA), and PLA. All listed performance aspects demonstrate that S‐bioplastic has excellent mechanical properties, thermal stability, and environmental sustainability, indicating that S‐bioplastic is a promising bioplastic and provides an effective solution to reduce plastic pollution.

### 2.2 Structural Characterization of S‐Bioplastic

**FIGURE 2:**
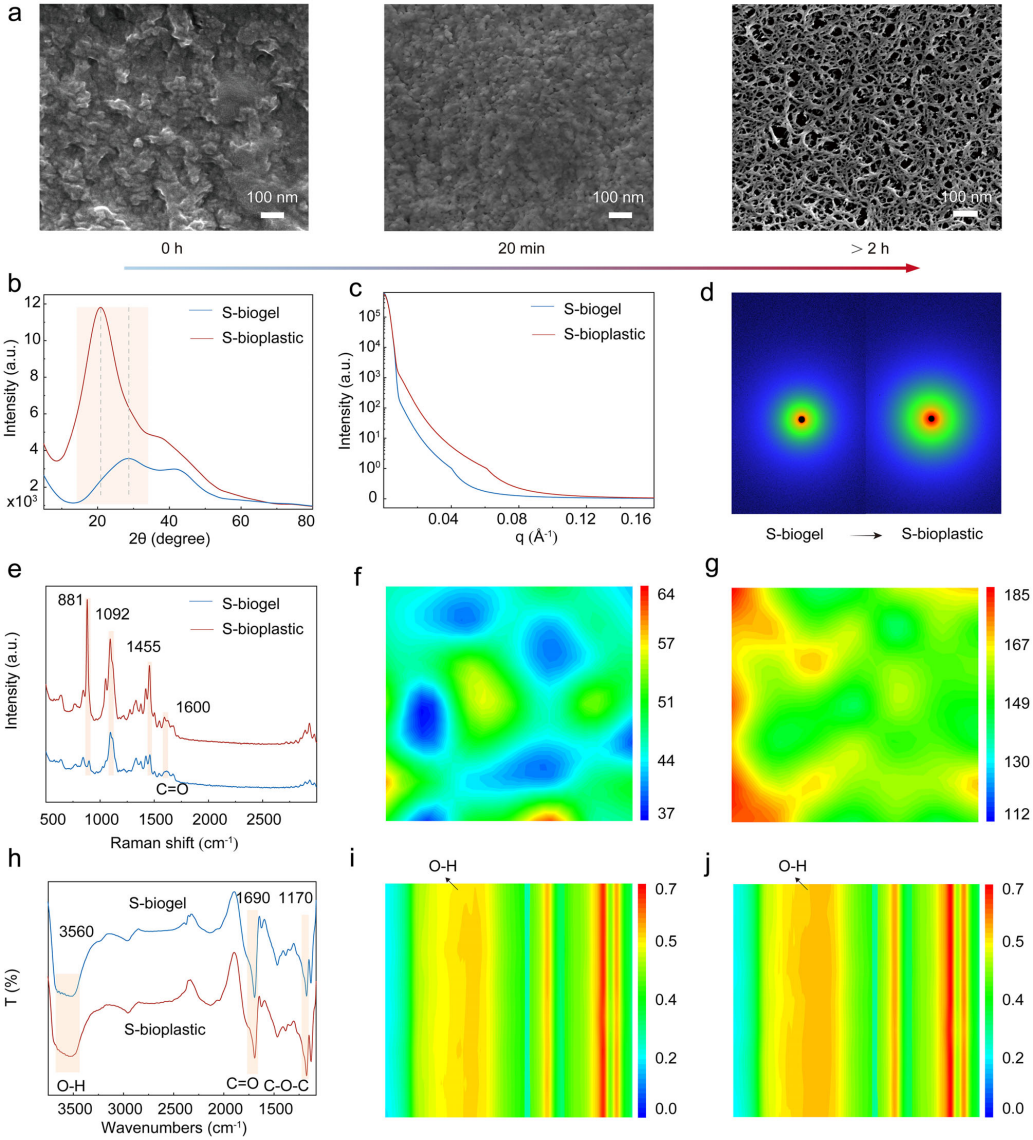
Structural characterization of S‐bioplastic. (a) SEM images of the S‐bioplastic during the ethanol stimulation process. (b) XRD pattern of S‐biogel and S‐bioplastic. (c) Investigating the SAXS curves of S‐biogel and S‐bioplastic. (d) Comparison of SAXS 2D patterns between S‐biogel and S‐bioplastic. (e) Raman spectra of S‐biogel and S‐bioplastic. (f) 2D Raman images of S‐biogel and (g)S‐bioplastic from C═O (about 1600 cm^−1^). (h) FTIR spectra of S‐biogel and S‐bioplastic. (i) 2D FTIR images of S‐biogel and (j) S‐bioplastic from O─H (about 3440–3700 cm^−1^).

Changes in the microstructure of materials can significantly affect their macroscopic physical properties. The micro‐morphologies of S‐biogel before and after ethanol stimulation were compared using a scanning electron microscope (SEM) (Figure 2a; Figure S5). With the extension of ethanol stimulation time, the S‐biogel gradually changed from a loose and microphase‐separated morphology to a dense one, and finally presented a dense and interwoven porous microstructure of S‐bioplastic. This enhanced structural morphology is the main reason for the improvement of its mechanical properties and plasticity (Figure S6). Further research was conducted on the structural changes of S‐bioplastic during the configuration process. X‐ray diffraction (XRD) analysis showed that compared with S‐biogel, S‐bioplastic exhibited sharper crystal diffraction peaks (Figure 2b), indicating that S‐bioplastic has ordered crystalline regions and a densely arranged microstructure. In addition, small‐angle X‐ray scattering (SAXS) and wide‐angle X‐ray scattering (WAXS) showed that compared with S‐biogel, S‐bioplastic displayed a stronger scattering signal (Figure 2c,d; Figure S7). These research results collectively indicate that under the stimulation and induction of ethanol, the supramolecular network of S‐biogel undergoes a self‐reinforcement process, forming denser and more ordered structural domains.

The molecular conformational changes of S‐biogel were analyzed using Raman spectroscopy and Fourier transform infrared spectroscopy (FTIR). As shown in Figure 2e, the characteristic peaks of S‐bioplastic belonging to bamboo cellulose (ring‐internal C─O─C, glycosidic bond C─O─C, and C─O) at 881 cm^−1^, 1092 cm^−1^, and 1455 cm^−1^ and the characteristic peak of C═O belonging to PAM at 1600 cm^−1^ were enhanced, this indicates that under the stimulation of ethanol, the bamboo cellulose molecular chains and PAM molecules are intertwined with each other, and the interaction between them is significantly enhanced. 2D Raman images (Figure 2f,g; Figure S8) show that the integral area of the characteristic peaks of S‐bioplastic is significantly higher than S‐biogel. This further confirms that the stimulation of ethanol causes the formation of an entangled structure between PAM and the molecular chains of bamboo cellulose. It is precisely this entangled structure that not only enhances the strength of S‐bioplastic, but also balances tension through slippage and disperses stress. This successfully resolves the traditional conflict between modulus and toughness in bioplastics.

In addition, in the Raman spectrum (Figure 2e), the Raman peak of the C═O group in S‐bioplastic exhibits a red shift, indicating that the entanglement between bamboo cellulose and PAM was accompanied by the enhancement of the H‐bond network. 1D FTIR (Figure 2h) showed that compared with S‐biogel, the O─H characteristic peak of S‐bioplastic was significantly broadened, reflecting the formation of strong H‐bonds between bamboo cellulose and PAM. The corresponding 2D FTIR images (Figures 2i,j) reveal that the region corresponding to O─H groups in S‐bioplastic exhibits darker colors, which further proves the trend of H‐bond enhancement, and the results are also consistent with XPS data analysis (Figure S9). All results indicate that the stimulation of ethanol not only enhances the H‐bond strength between bamboo cellulose molecules and PAM, but also promotes their mutual entanglement and forms a stable supramolecular [43, 44].

### 2.3 Mechanical Properties of S‐Bioplastic

**FIGURE 3:**
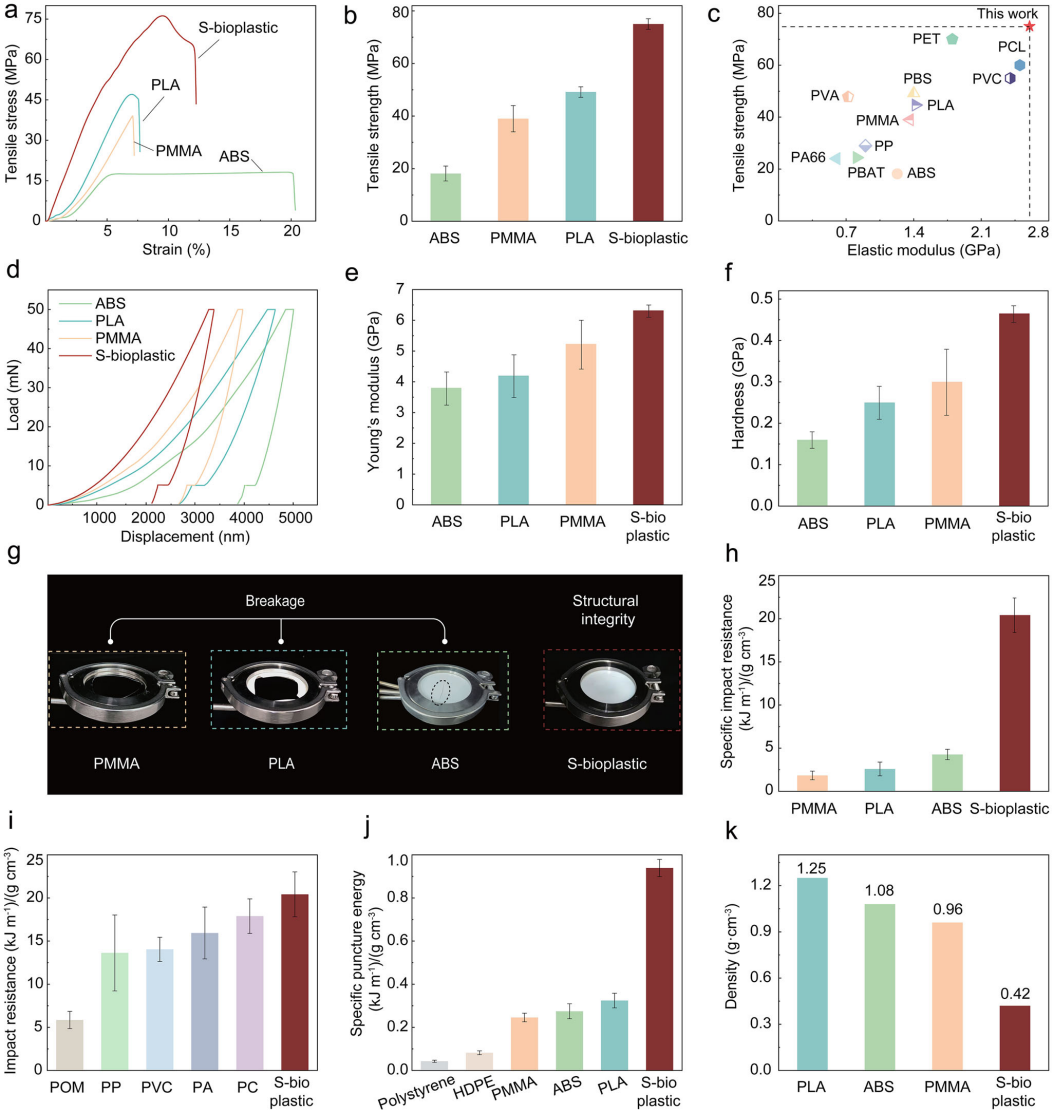
Comparison of mechanical properties between S‐bioplastics and commercial plastics (ABS, PMMA, and PLA). (a) Tensile stress‐strain curves (b) Tensile strength. (c) Comparison of the mechanical performance of S‐bioplastic and some widely used plastics. (d) load‐displacement curves by nanoindentation. (e) Young's modulus by nanoindentation. (f) Hardness by nanoindentation. (g) Free‐fall impact performance test of S‐bioplastic digital photos and (h) impact resistance. (i) Impact resistance (j) Puncture resistance. (k) Densities of commercial plastics compared with that of the S‐bioplastic.

The mechanical properties of S‐bioplastic were evaluated, including tensile strength, hardness, impact resistance, and puncture resistance. Comparisons were made with commercial plastics such as ABS, PMMA, and PLA. As illustrated in Figure 3a, the stress‐strain curve of S‐bioplastic did not break immediately after reaching the yield peak, but showed a slow stress drop process, and finally broke. This slow decline process indicates that the expansion of cracks is hindered to a certain extent by the surrounding dense network, and it is necessary to continuously consume energy to destroy more H‐bonds [45, 46]. The tensile strength of S‐bioplastic is measured at 76.2 ± 2.2 MPa, substantially higher than that of ABS (18.16 ± 2.84 MPa), PMMA (39.1 ± 1.36 MPa), and PLA (49.16 ± 2.1 MPa) plastics (Figure 3b). Compared with many plastics, S‐bioplastic also has better tensile strength and elastic modulus (Figure 3c) [47]. This excellent performance of both high strength and high modulus can be attributed to the formation of a self‐reinforcing supramolecular network: tightly arranged cellulose molecular chains form a strong molecular framework [48], significantly improving the stiffness of the material, while PAM molecules, through entanglement, synergize with cellulose molecules to achieve multi‐scale stress dispersion, greatly enhancing the toughness of the material.

Nanoindentation and nano‐scratch tests (Figure 3d) showed that under the same load, the indentation depth of S‐bioplastic was lower. The test results indicated that the Young's modulus and hardness of S‐bioplastic were 6.32 ± 0.2 GPa and 0.46 ± 0.02 GPa, respectively, which were both higher than those of ABS, PMMA, and PLA plastics (Figure 3e,f). In addition, the S‐bioplastic exhibited excellent scratch resistance; under a thrust force of 500 mN, its scratch width was only 52.1 ± 1 µm (Figure S10a). This enables the S‐bioplastic to have significant scratch hardness and plastic deformation rate (Figure S10b,c), further highlighting its excellent structural performance.

The impact resistance is another excellent characteristic of the S‐bioplastic. In the free‐fall impact test, a 256 g iron ball was dropped from a height. PMMA, PLA, and ABS plastics all suffered structural damage, while the S‐bioplastic maintained structural integrity and absorbed the impact force with minimal damage (Figure 3g; Figure S11). This indicates that this self‐reinforcing supramolecular network can effectively dissipate impact energy, thereby maintaining its structural integrity. We demonstrated that the impact resistance of S‐bioplastic is significantly higher than that of PMMA, PLA, and ABS plastics (Figure 3h), reaching 20.4 ± 2.1 kJ·m^−^^1^·(g·cm^−^^3^)^−^^1^.

Even when compared to the impact resistance of other commercial plastics, S‐bioplastic still has significant advantages (Figure 3i) [49]. Meanwhile, S‐bioplastic also exhibits excellent puncture resistance with a puncture‐specific energy absorption of 0.94 ± 0.04 kJ·m^−^^1^·(g·cm^−^^3^)^−^^1^, which is 11 to 21 times higher than polystyrene and high‐density polyethylene (HDPE), and also significantly higher than commercial plastics (Figure 3j; Figure S12). These results further highlight that S‐bioplastic has high strength, low density (Figure 3k), and lightweight properties, making it more advantageous compared to various traditional plastic materials.

### 2.4 Thermal Stability of S‐Bioplastic

**FIGURE 4:**
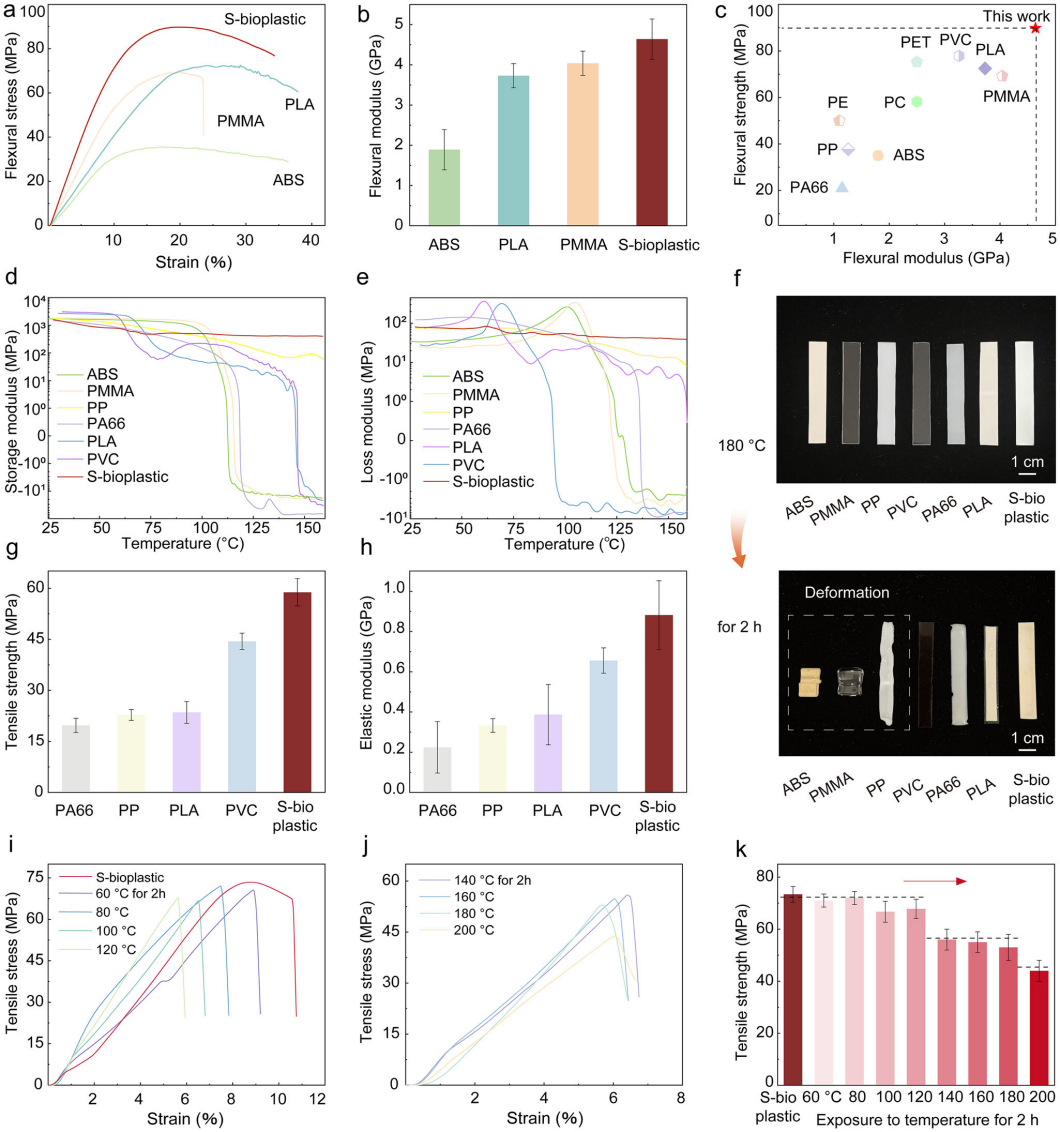
Mechanical and thermal properties of bioplastic. (a) Flexural stress‐strain curves of S‐bioplastic and (b) flexural modulus, compared with ABS, PMMA, and PLA. (c) Comparison of flexural strength versus modulus for S‐bioplastic with some widely used plastics. (d) Comparison of the storage modulus and (e) loss modulus of S‐bioplastic with that of several commercial plastics. (f) Optical images, (g) tensile strength, and (h) elastic modulus of S‐bioplastic at 180°C for 2 h, compared with various commercial plastics. (i–k) Comparison of the mechanical properties of S‐bioplastic under different high temperature conditions.

For polymer materials, maintaining the stability of their structure and performance under extreme temperature conditions is crucial, and the bending performance of the material is a key indicator for quantifying the impact of thermal stability on actual mechanical performance. As shown in Figure 4a, S‐bioplastic's flexural stress increases with strain until peaking, then exhibits yielding behavior. At this stage, the entangled cellulose‐PAM structure dissipates external energy through slipping and H‐bond breaking, enabling slow stress release and avoiding brittle fracture from stress concentration. Thus, S‐bioplastic demonstrates excellent ductility and toughness, undergoing large deformation without breaking. Its flexural strength reaches 89.8 ± 2.1 MPa and flexural modulus 4.7 ± 0.5 GPa, significantly outperforming common commercial plastics (Figure 4b,c). Additionally, S‐bioplastic shows good fatigue resistance. In 50 cyclic tensile tests, residual strain after each cycle gradually decreases with increasing cycles, indicating the internal structure stabilizes during repeated loading, demonstrating excellent durability and fatigue resistance (Figure S13).

Due to the constraints imposed by the self‐reinforcing supramolecular network on the thermal motion of molecular chains, S‐bioplastic demonstrates superior thermal stability compared to commercial plastics. Thermogravimetric analysis (TG) shows a high decomposition temperature of 245°C (Figure S14a). Differential scanning calorimetry (DSC) curves (Figure S14b) reveal no discernible endothermic or exothermic behavior during heating‐cooling cycles, indicating exceptional structural stability from −50°C to 150°C.

Dynamic mechanical analysis (DMA) showed that the S‐bioplastic modulus remained stable in the range of 25–150°C, and the storage modulus was always greater than the loss modulus (Figure 4d,e; Figure S15). This indicates that the material is in a solid state within this temperature range, and the chain segment motion of the molecular chain is strongly restricted by the self‐reinforcing supramolecular network, and no obvious glass transition or softening occurs. S‐bioplastic also exhibits a low coefficient of thermal expansion of 4.42 × 10^−5^ K^−1^ in this temperature range (Figure S16), which further underscores its excellent dimensional stability and meets high‐temperature material requirements for engineering applications. Hot plate experiments directly show S‐bioplastic maintains a stable form at 180°C for 2 h, while commercial plastics soften and deform (Figure 4f). Notably, after high‐temperature treatment, it retains tensile strength of 58.8 ± 3.7 MPa and elastic modulus of 0.9 ± 0.17 GPa (Figure 4g,h). This dense hydrogen bond network and the entanglement of PAM molecular chains jointly restrict the relaxation of molecular chains at high temperatures, enabling S‐bioplastic to maintain high mechanical properties at high temperatures and further demonstrating excellent high‐temperature resistance.

We further studied the mechanical properties of S‐bioplastic under different high‐temperature conditions. As shown in Figure 4i,j, with the increase of temperature, the plastic can still maintain excellent mechanical properties. Even after exposure to 200°C for 2 h, it still has a significant tensile strength of about 48 MPa (Figure 4k), highlighting its structural stability. The above thermal properties indicate that S‐bioplastic has excellent thermal stability and is expected to become a more reliable and efficient alternative to existing plastics in high‐temperature application scenarios.

### 2.5 Low‐Temperature Resistance and Plasticity

**FIGURE 5:**
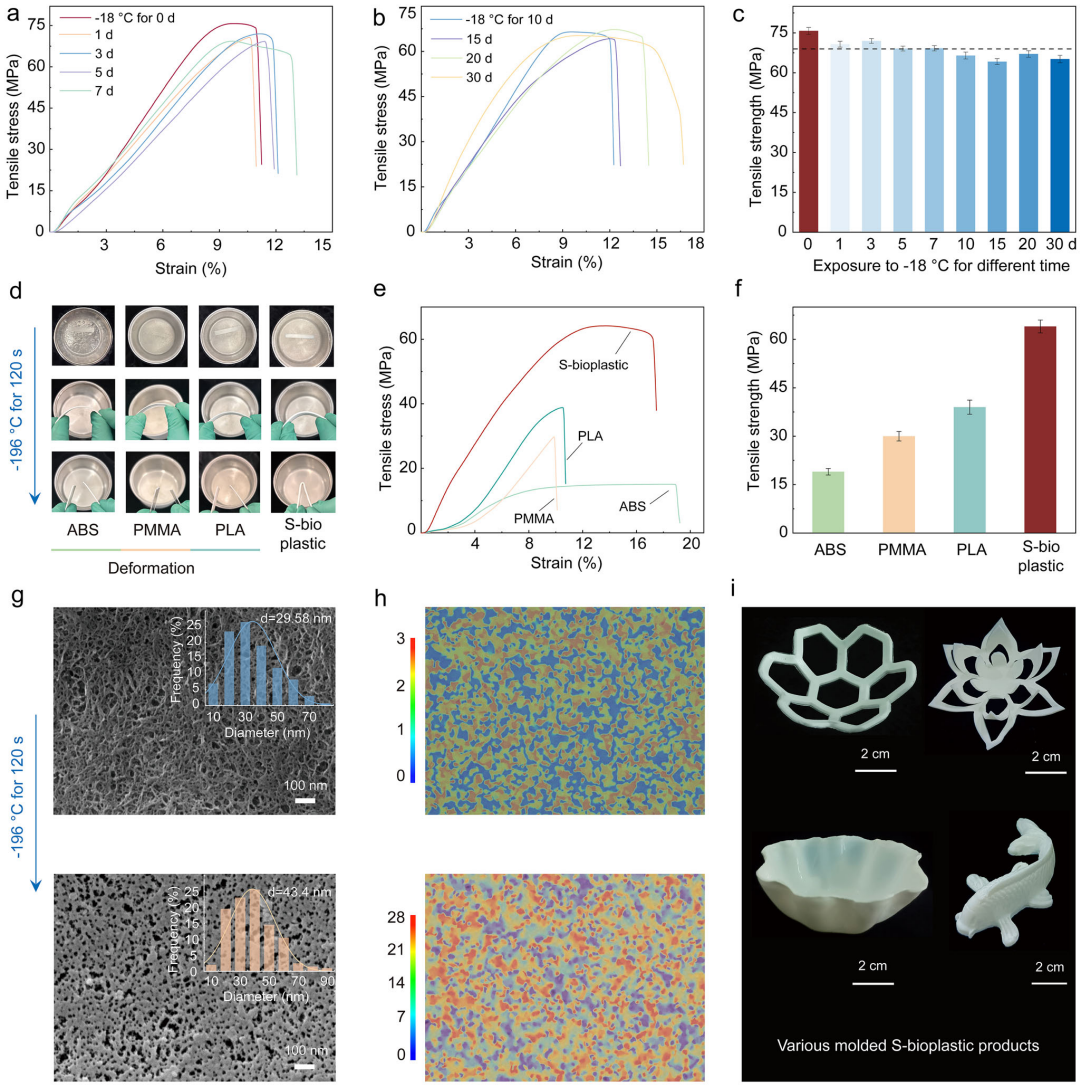
Low‐temperature resistance and formability of S‐bioplastic. (a–c) The mechanical properties of S‐bioplastic exposed at −18°C for different times were compared. (d) Optical images of S‐bioplastic, ABS, PMMA, and PLA after being placed in liquid nitrogen at −196°C for 120 s, and (e, f) comparison of mechanical properties. SEM images (g) and SDOF images (h) of S‐bioplastic before and after exposure to liquid nitrogen at −196°C for 120 s. (i) Various molded S‐bioplastic products.

Beyond exceptional mechanical strength and thermal stability, S‐bioplastic also exhibits low‐temperature resistance. Systematic evaluation of low‐temperature applicability involved testing mechanical properties after exposure to −18°C for varying durations. As shown in Figure 5a,b, S‐bioplastic maintained stable mechanical performance with increasing exposure time at −18°C, retaining high tensile strength of 65.17 ± 1.4 MPa even after 30 days (Figure 5c). Further research has been conducted on the stability at extremely low temperatures, when ABS, PMMA, PLA, and S‐bioplastic were exposed to liquid nitrogen (−196°C) for 120 s, bending tests showed ABS, PMMA, and PLA fractured, while S‐bioplastic demonstrated excellent flexibility (Figure 5d). Notably, after extreme low‐temperature treatment, S‐bioplastic retained tensile strength of 64.2 ± 1.8 MPa (Figure 5e,f), confirming structural integrity and stability without obvious damage or failure, indicating excellent durability in extreme low‐temperature environments.

We explore the intrinsic mechanism of the low‐temperature resistance of S‐bioplastic at the microstructural level. SEM observations reveal that S‐bioplastic exhibited an intertwined porous network structure with an average pore size of about 29.58 nm. After 120 s exposure to −196°C liquid nitrogen, the porous network morphology was retained, but the average pore size expanded to about 43.4 nm (Figure 5g). Super Depth of Field Microscopy further confirmed significantly increased surface height differences of S‐bioplastic after liquid nitrogen treatment (Figure 5h), consistent with pore size expansion and reflecting coordinated microscale (pore size) and macroscale (surface height difference) changes. Such multi‐scale coordination effectively alleviates stress concentration during rapid low‐temperature cooling, enabling retention of 85% tensile strength and 70% elastic modulus after −196°C treatment (Figure S17). These results demonstrate that S‐bioplastic exhibits remarkably low‐temperature adaptability, effectively suppressing low‐temperature‐induced brittle failure.

Plasticity is essential for S‐bioplastic to meet product shape requirements, enable large‐scale processing, and facilitate practical applications. Similar to commercial plastics, S‐bioplastic can be formed through coating and casting methods. These features allow us to design and manufacture complex and elaborate 3D special‐shaped structures according to the needs of different scenarios, and processed into large‐sized products (Figure 5i; Figure S18). Notably, the S‐bioplastic after plasticization exhibits excellent load‐bearing capacity. A 1.6 g S‐bioplastic with a triangular 3D shape can support the weight of a 70‐kilogram adult male, which is equivalent to 43,000 times its own weight (Figure S19). The characteristics of strong environmental tolerance, stable performance, high plasticity, and ease of large‐scale production make S‐bioplastic an ideal choice for lightweight and sustainable plastics.

Our research further evaluated the tolerance of S‐bioplastic to common solvents (Figure S20). It was observed that after 30 days of immersion, S‐bioplastic maintained its original shape and stiffness without any noticeable morphological changes, indicating its good resistance to the most common solvents. In addition, the water sensitivity issue of S‐bioplastic can be significantly improved through simple PLA modification [50]. After modification, the swelling of S‐bioplastic‐PLA in water was significantly suppressed, the water contact angle increased (Figure S21), and the mechanical properties remained relatively stable (Figure S22). The above results indicate that PLA modification has no significant effect on the overall performance of S‐bioplastic, which confirms that hydrophobic modification of S‐bioplastic with PLA is a simple and effective strategy for improving water resistance.

### 2.6 Recyclability, Biocompatibility, Degradability, and Economic Feasibility

**FIGURE 6:**
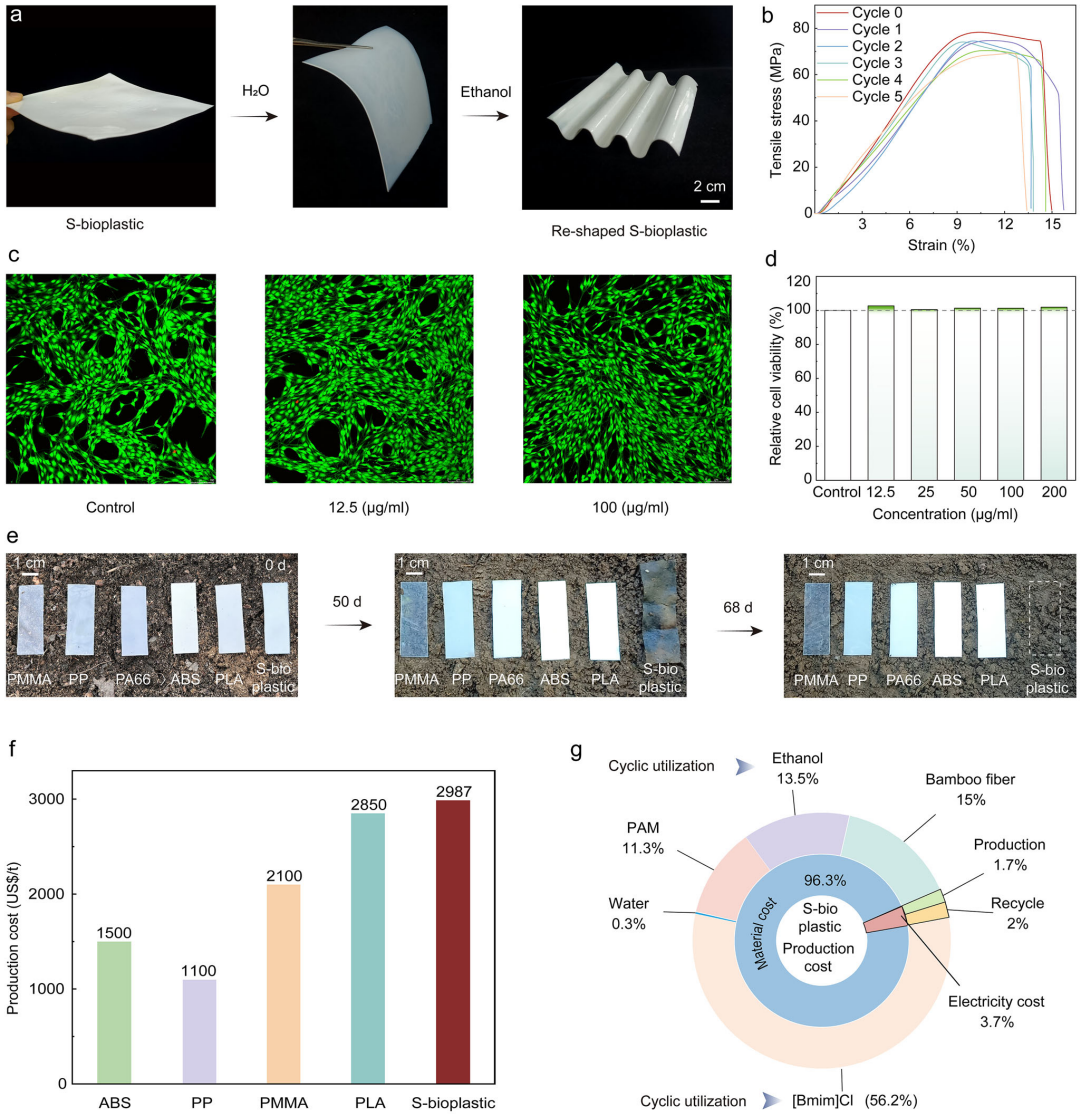
Recyclability, biocompatibility, biodegradability, and economic feasibility of S‐bioplastic. (a) Recyclability. (b) The stress‐strain curve of recycled S‐bioplastic. (c) Investigating the cell viability of S‐bioplastic in different cell suspension concentrations. (d) Live staining confocal images of skin tissue cells co‐cultured with different concentrations of S‐bioplastic for 24 h. (e) The biodegradability tests of S‐bioplastic and commercial plastics under natural soil. (f) Comparison of production costs between S‐bioplastics and some commercial plastics. (g) Production cost analysis of S‐bioplastic.

S‐bioplastic can be recycled through water‐responsive dynamic hydrogen bonds. As shown in Figure 6a, recycled S‐bioplastic can be restored to flexible S‐biogel through water immersion and easily re‐shaped into products under ethanol stimulation. Impressively, the strength retention rate after the first recycling reaches 95.4%, and after 5 cycles, S‐bioplastic still retains 88.3% of its tensile strength and 88.9% of its elastic modulus, demonstrating excellent performance recovery and reprocessing capabilities (Figure 6b; Figure S23 and Table S1). Additionally, ionic liquid ([Bmim]Cl) and ethanol used in preparation can be recycled by collecting mixed solutions and recovering solvents through evaporation‐condensation, further highlighting sustainable utilization potential.

Cytotoxicity analysis evaluated S‐bioplastic's biocompatibility. As shown in Figure 6c,d, cell survival rates exceeded 100% across various concentrations, confirming excellent biocompatibility. S‐bioplastic also exhibits impressive biodegradability in natural soil (Figure 6e): when buried at 10 cm depth, it swelled after 50 days, showed significant surface erosion and cracking, and achieved complete morphological disintegration within 68 days, while commercial plastic retained original morphology. Effective biodegradation in natural soil addresses persistent plastic pollution, highlighting its great potential as an environmentally friendly plastic alternative.

Techno‐economic analysis (TEA) evaluated cost differences between S‐bioplastic and commercial plastics. Results show S‐bioplastic production costs are higher than petrochemical plastics (PMMA, ABS, PP) and PLA (Figure 6f; Table S2). Since electricity costs account for only 3.7%, this low‐energy consumption production process has significant advantages (Figure 6g; Table S3). Despite slightly higher production costs than PLA, S‐bioplastic's excellent mechanical properties, superior environmental tolerance, good processability, and lifecycle environmental friendliness enhance market competitiveness and application prospects in the bioplastic sector.

## 3 Conclusion

This study introduces a design for a self‐reinforcing supramolecular network that employs bamboo cellulose as the molecular framework, coupled with in situ polymerization of PAM molecules. By leveraging an ethanol‐stimulated supramolecular network to enhance its self‐reinforcing behavior, we successfully developed S‐bioplastic, which exhibits remarkable mechanical attributes: a tensile strength of 76.2 ± 2.2 MPa, an impact energy of 20.4 ± 2.1 kJ·m^−^^1^·(g·cm^−^^3^)^−^^1^, and a flexural modulus of up to 4.7 ± 0.5 GPa. Furthermore, S‐bioplastic demonstrates impressive thermal stability, exceeding 180°C, and maintains low‐temperature resistance down to ‐196°C, surpassing both petrochemical plastics (such as ABS and PMMA) and PLA bioplastics. Additionally, S‐bioplastic exhibits good plasticity, allowing it to be shaped through ethanol stimulation, thus facilitating the large‐scale production of complex 3D geometric structures and sizable products in various environmental conditions, which enhances its practical application potential.

Moreover, S‐bioplastic demonstrates notable recyclability, retaining 95.4% of its original mechanical strength after reprocessing, along with excellent biocompatibility and complete biodegradation in natural soil within 68 days. An economic assessment indicates that the production of S‐bioplastic is characterized by low energy consumption and manageable costs, reflecting its strong market competitiveness. In summary, the exceptional properties of S‐bioplastic position it as a viable and sustainable alternative to petroleum‐based plastics.

## 4 Experimental Section

### 4.1 Materials

Bamboo stems, 1‐Butyl‐3‐methylimidazolium chloride ([Bmim]Cl), acrylamide (AM), ammonium persulfate (APS), N,N′‐methylenebis(acrylamide) (BIS), and anhydrous ethanol were purchased from Aladdin (China). Formic acid (AC), acetic acid (HAC), tetrahydrofuran (THF), ammonia (NH_3_·H_2_O), methanol (ME), acetone (ACE), and N, N‐dimethylformamide (DMF) were purchased from Fuyu Chemical (China).

### 4.2 Preparation of Bamboo Cellulose

Bamboo cellulose was prepared by selectively removing the lignin and hemicellulose from the bulk bamboo. Specifically, bamboo chips were delignification with 0.07 m NaClO_2_ solution (add an appropriate amount of acetic acid) at 75°C for 6 h, and then washed three times with deionized water to remove residual chemicals. Subsequently, the obtained bamboo samples were placed in a 0.05 m KOH solution at 90°C for 2 h to remove hemicellulose. After the reaction, the solution was rinsed with deionized water several times until it became neutral. After suction filtration and drying, the purified bamboo cellulose was obtained.

### 4.3 Preparation of Bamboo Cellulose Hydrogel

Approximately 25 g of [Bmim]Cl ionic liquid was mixed with 4.41 g of bamboo cellulose at 85°C for 6 h, resulting in a bamboo cellulose ionic gel. The cellulose ionic gel was uniformly spin‐coated onto a smooth glass plate and placed in a circulating drying oven at 85°C for at least 12 h to remove bubbles. Afterward, the glass plate containing the cellulose ionic gel system was removed and kept at room temperature to adjust the humidity for ≈12 h, ultimately preparing a cellulose ionic gel. This cellulose ionic gel was torn off the glass plate and then placed in deionized water for ≈6 h to obtain a flexible bamboo cellulose hydrogel.

### 4.4 Preparation of Polymer Monomer Solution

The mixed‐monomer solution used in constructing a PAM molecular network was prepared as follows: 80 g of AM and 600 g of deionized water were fully dissolved and stirred magnetically at room temperature for 20 min. Subsequently, 0.4 g of APS was added as an initiator, and 0.08 g of BIS was added as a cross‐linking agent.

### 4.5 Preparation of S‐Biogel

The obtained bamboo cellulose hydrogel was immersed in the polymer molecular solution for more than 12 h at room temperature, followed by in situ polymerization for 8 h in a vacuum drying oven at 65°C. An S‐biogel with supramolecular networks was then prepared by stripping the surface polyacrylamide.

### 4.6 Preparation of S‐Bioplastic

The S‐biogel was immersed in an ethanol solution for stimulation treatment over a period of 12 h. Following this induction process, the S‐bioplastic can be obtained and left in open air for 12 h to facilitate the evaporation of ethanol, bamboo cellulose content of ≈57.37 wt.%, and PAM content of ≈42.63 wt.% in dry.

### 4.7 Molding Process of S‐Bioplastic

The S‐biogel was positioned in the designated mold and then submerged in an ethanol solution for 12 h to obtain a mold‐shaped plastic.

### 4.8 Fabrication of S‐Bioplastic‐PLA

All reagents and raw materials are commercially available. In a typical preparation process, 15 g polylactic acid (PLA) (Rhawn, Mw∼110000) was first placed in 100 mL in a crucible and heated in a preset high temperature oven at 190°C for 1 h. Then, the S‐bioplastic sample was immersed in the molten PLA slurry for 5 s and quickly taken out. Finally, the samples were cooled down at room temperature to form a thin PLA film on the surface, and then the S‐bioplastic‐PLA sample was obtained.

## Author Contributions

D.Z. supervised the project. J.L. carried out most of the experiments. G.J. and S.Z. participated in mechanical performance analysis and contributed to microscopic morphology analysis. J.L. wrote the paper. D.Z., H.Y., and M.W. discussed the results and designed the mechanism. D.Z., M.W., and H.Y. collectively reviewed the paper. All authors read and approved the final submitted manuscript.

## Conflicts of Interest

The authors declare no conflicts of interest.

## Supporting information




**Supporting File**: advs74426‐sup‐0001‐SuppMat.docx.

## Data Availability

The data that support the findings of this study are available on request from the corresponding author. The data are not publicly available due to privacy or ethical restrictions.
