# Supplementary material for: Stimulus‐Induced Self‐Reinforcement in Supramolecular Bamboo Plastics toward Mechanical Robustness and Programmable Shapeability
Source: Adv Sci (Weinh). 2026 Feb 15;13(24):e74426. doi: 10.1002/advs.74426 (PMC13116100; doi:10.1002/advs.74426)
Supplement: Supplementary file 1 — Supporting File: advs74426‐sup‐0001‐SuppMat.docx. [file ADVS-13-e74426-s001.docx]

**SUPPORTING INFORMATION**

**I. Supplementary Method**

**Characterization.** The Scanning electron microscopy (SEM) microstructure of samples was characterized by a JSM-IT800 microscope (JEOL, Tokyo, Japan) at an operating voltage of 10 kV. The XRD pattern of sample was measured by a D/max 2200 X-ray diffractometer (Rigaku, Tokyo, Japan) equipped with Ni-filtered Cu-Kα radiation (λ = 0.154 nm). The samples were scanned within 5-90° 2θ at 30 kV and 30 mA with a scanning rate of 5°·min^-1^. The small-angle X-ray scattering (SAXS) of sample was measured using an SAXS mc^2^ instrument with Cu-Kα X-ray radiation and a wavelength of 0.154 nm (Anton Paar, Graz, Austria). The sample-to-detector distance was 2658.5 mm and exposure time 5 min. Grazing-incidence wide-angle X-ray scattering (GIWAXS) of samples was measured using a BXeuss 3.0 SAXS (Xenocs, France). Raman spectroscopy was performed using a high-resolution microscopic confocal Raman spectrometer (HR Evolutio) equipped with a 785 nm laser. The high-resolution microscopic confocal Raman spectrometer (HR Evolutio) was utilized to perform spatial Raman mapping. The wavelength of the excitation laser was 785 nm and the Raman mappings of the sample surface to the strength of the peak region of the characteristic peak (scan range 35 μm × 35 μm) were obtained. The FTIR spectra of samples were obtained by a Nicolet IS50 FTIR instrument (Thermo Fisher Scientific Inc., Waltham, MA, USA). All spectra were measured in ATR mode, with data recorded in the range of 500-4000 cm^-1^. The FTIR imaging of the sample was obtained by point scanning (scanning range 50 μm × 50 μm) of a confocal infrared microscope Confocal Infrared Microscope IR (Thermo Fisher Scientific Inc., Nicolet iN10). The acquisition time of each point is 3 s. X-ray photoelectron spectroscopy (XPS) spectra were obtained by the Shimadzu Model Axis Supra (Japan) equipped with monochromator Al target (λ = 0.05 eV. The thermogravimetry (TG) and differential thermal analysis (DTA) of samples were characterized using a STA6000 thermal gravimetric analyzer (Perkin Elmer, Massachusetts, USA). The DSC curve of sample was measured by DSC 3+ (Mettler Toledo, CH) with a temperature range of -50−150 °C. The heating rate and cooling rate both were 5 °C·min^-1^.

**Mechanical properties test**

The tensile properties and elasticity modulus of the samples were tested by microcomputer-controlled electronic universal testing machine (Sansi Zongheng, UTM2503). The test samples size was 5 cm (length) × 1 cm (width) × 1-1.2 mm (thickness), and each group had three parallel sample strips.

For the impact resistance test of iron ball, we used a metal iron ball (256 g) as a free-falling impactor, the velocity of iron ball just touching sample was calculated by free-fall formula, *ν*_0_ = (2*hg*)^1/2^, where *h* is the free-fall distance (m) and *g* is the acceleration of gravity (9.8 m s^−2^). We adjusted *h* until the iron ball broke the samples. This was repeated five times for each material. The energy absorption of destruction of the test samples was defined by loss of kinetic energy after iron ball attacking samples. The impact resistance for specific energy absorption is calculated by (*mν*_0_^2^)/2*dρ*, where *m* is the mass of iron ball, *d* is the thickness of sample, *ρ* is the density of sample and *ν*_0_ is the velocity of iron ball touching sample. Puncture resistance test was carried out with 80.5 g steel needle.

**Investigating the scratch resistance**

According to the ASTM G171-03 (2009) scratch hardness test method, a linear reciprocating friction and wear tester (UMT-3, USA) was used to evaluate the scratch resistance of samples. The test was performed by applying a normal load on the diamond ball tip indenter (tip radius of 20 μm) and moving the surface of the sample (20 × 20 × 1 mm) horizontally relative to the indenter at a constant speed. The width of the beginning, middle and last three parts of each scratch is measured using a nano-detection microscope (LEXT OLS4500, Japan).

**Nanoindentation test**

Nanoindentation testing was conducted using an Anton Paar UNHT (Ultra Nano Hardness Tester) system to characterize the hardness and Young's modulus of micro/nano-scale hard thin film materials and wear-resistant coatings. Specimens with dimensions of 2 × 2 cm^2^ and 1 mm thickness were analyzed under a maximum applied load of 50 mN, following established protocols for nanomechanical property evaluation of surface-engineered materials.

**Dynamic thermomechanical analysis (DMA)**

The dynamic viscoelastic properties of samples at varying temperatures were analyzed by DMA test. The DMA analysis was performed on a TA Q800 analyzer (TA Instruments, USA). The temperature ramp was from -80 to 150 °C at a frequency of 1 Hz and a heating rate of 5 °C/min, with a frequency sweep range from 0.1 to 100 Hz.

**Coefficient of thermal expansion (CTE)**

The CTE for the samples was determined using a TEA-3 Thermal expansion coefficient analyze (JouleYacht). The sample size is about 15 mm × 15 mm and tested over a temperature range of 25 to 150 °C.

**Cytotoxicity test for human fibroblasts**

The cytotoxicity of S-bioplastic extract liquid was evaluated using the CCK-8 assay. Extract liquids of S-bioplastic at various concentration (10, 25, 50, 100, and 200 μg/mL) were prepared by immersing them into complete media 24 h after sterilizing under UV irradiation. NHDF cells (4 x 10^3^ cell/well) were first seeded in 96-well plates and incubated with DMEM for 24 h (37 ℃, 5% CO_2_). After removing the culture medium, the unattached NHDF cells were rinsed with PBS buffer solution. Then, extract liquid with diverse concentrations was incubated with NHDF cells for another 24 h. Finally, 10 μL of a CCK-8 solution was added to wells containing cells and incubated together for 2 h. The cells treated without S-bioplastic extract were applied as a blank control. The absorbance value at 450 nm was measured using an enzyme immunoassay analyzer.

Cell viability = $\left[ \frac{\text{As}\text{-}\text{Ab}}{\text{Ac}\text{-}\text{Ab}} \right]\text{×100\%}$

Among them, *As* is the test hole absorbance, *Ac* is the control hole absorbance, *Ab* is the white hole absorbance.

The cytotoxicity test was conducted using the live/dead staining to detect the viability changes of NHDF cells. Extract liquids of S-bioplastic at various concentration (10, 200 μg/mL) were prepared by immersing them in complete media 24 h after sterilizing under UV irradiation. NHDF cells (2 x 10^4^ cell/well) were first seeded in confocal dish and incubated with DMEM for 24 h (37 ℃, 5% CO_2_). After removing the culture medium, the unattached NHDF cells were rinsed with PBS buffer solution. Then, extract liquid with diverse concentrations was incubated with NHDF cells for another 24 h. Before staining, the adherent cells were gently washed with PBS, the supernatant was removed, and the active esterase contained in the petri dish was removed. Add enough solution (2 μM Calcein-AM and 8 μM PI) and the cells were incubated at room temperature for 30-45 min away from light. Sucking out the dye work solution to terminate incubation. The cells treated without S-bioplastic extract were applied as a blank control. The stained cells were observed under confocal microscope.

**Biodegradability test**

To assess the biodegradability of various materials including ABS, PP, PA66, PMMA, PLA and S-bioplastic, samples were prepared with dimensions of 50 × 15 × 1 mm and subsequently interred at a depth of 10 cm in natural soil. Periodically, these specimens were retrieved for evaluation, and their morphological alterations were recorded utilizing digital photography.

**II. Supplementary Figures**


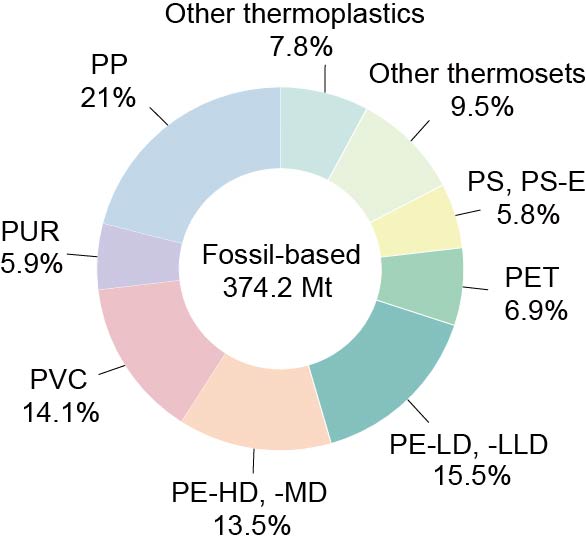


**Figure S1.** Proportion of fossil-based plastic production.

**
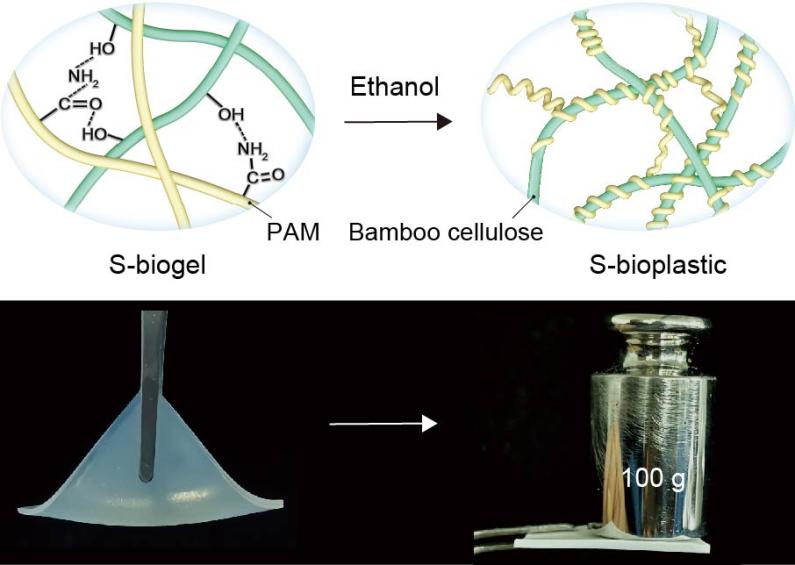
**

**Figure S2.** Supramolecular network structure design strategy of S-bioplastic.


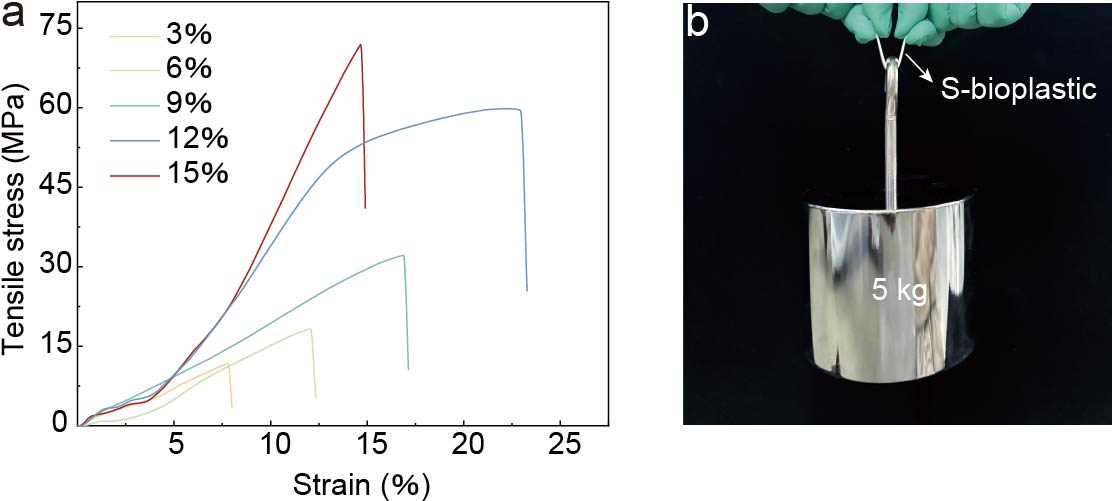


**Figure S3.** The mechanical tensile properties of S-bioplastic prepared with different bamboo cellulose concentrations were compared.


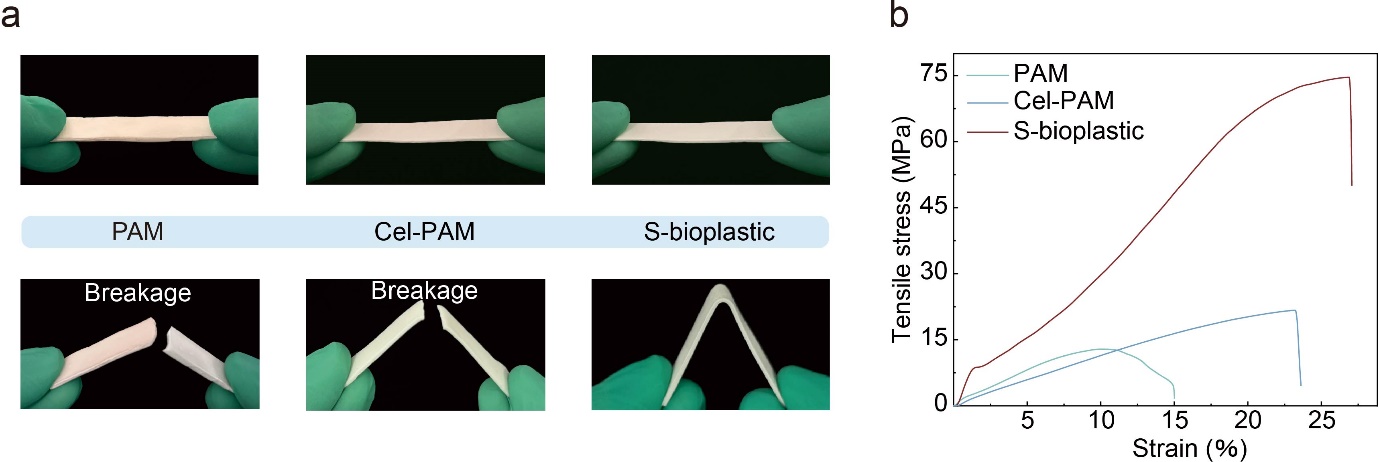


**Figure S4.** Compared with polyacrylamide (PAM) and wood cellulose/polyacrylamide (Cel/PAM), S-bioplastic exhibits good flexibility (a) and significant strength (b).


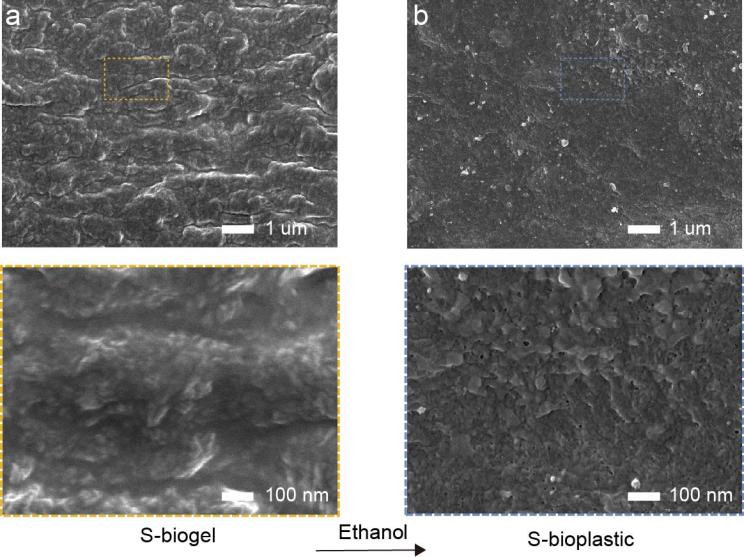


**Figure S5.** SEM cross-sectional images of S-biogel (a) and S-bioplastic (b).

**
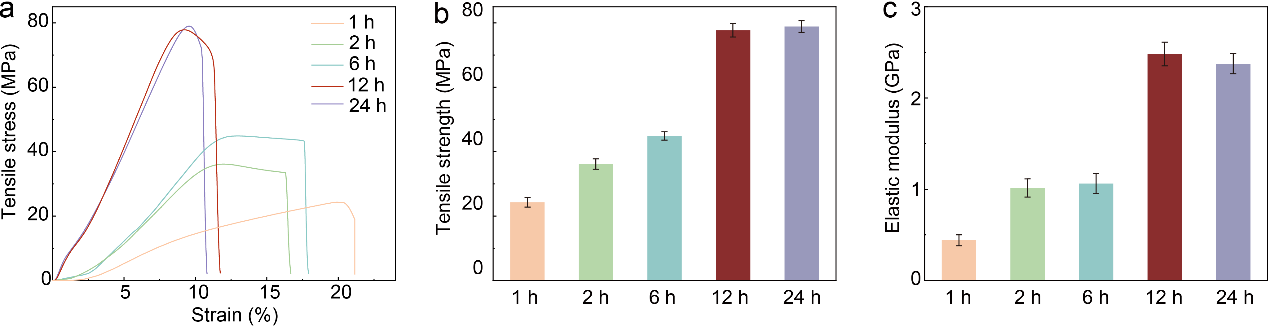
**

**Figure S6.** The effect of ethanol stimulation time on the mechanical properties of S-bioplastic. (a) Stress-strain curve. (b) Tensile strength. (c) Elastic modulus.


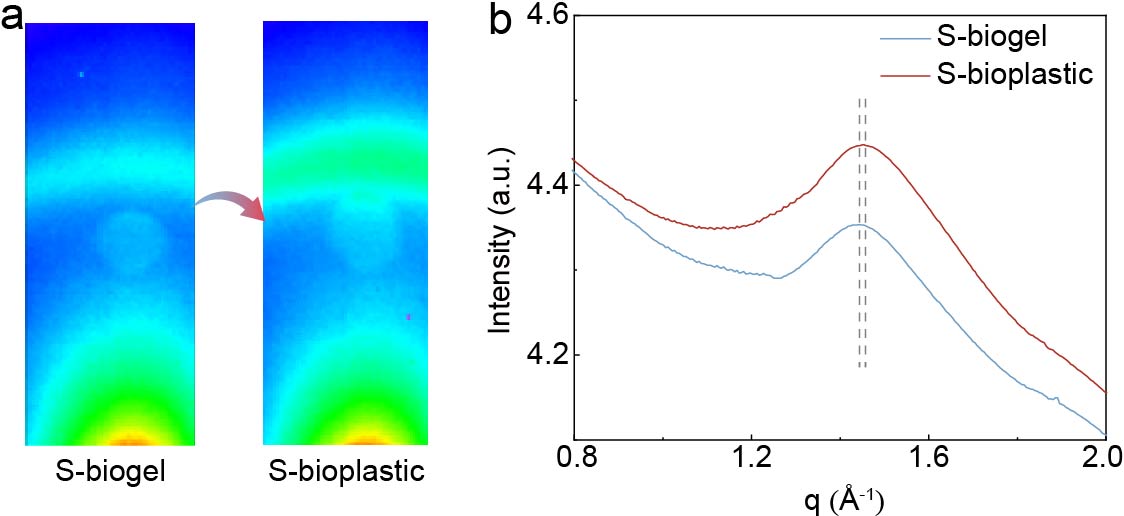


**Figure S7.** (a) GI-WAXS 2D patterns of S-biogel and S-bioplastic. (b) GI-WAXS curves of S-biogel and S-bioplastic

**
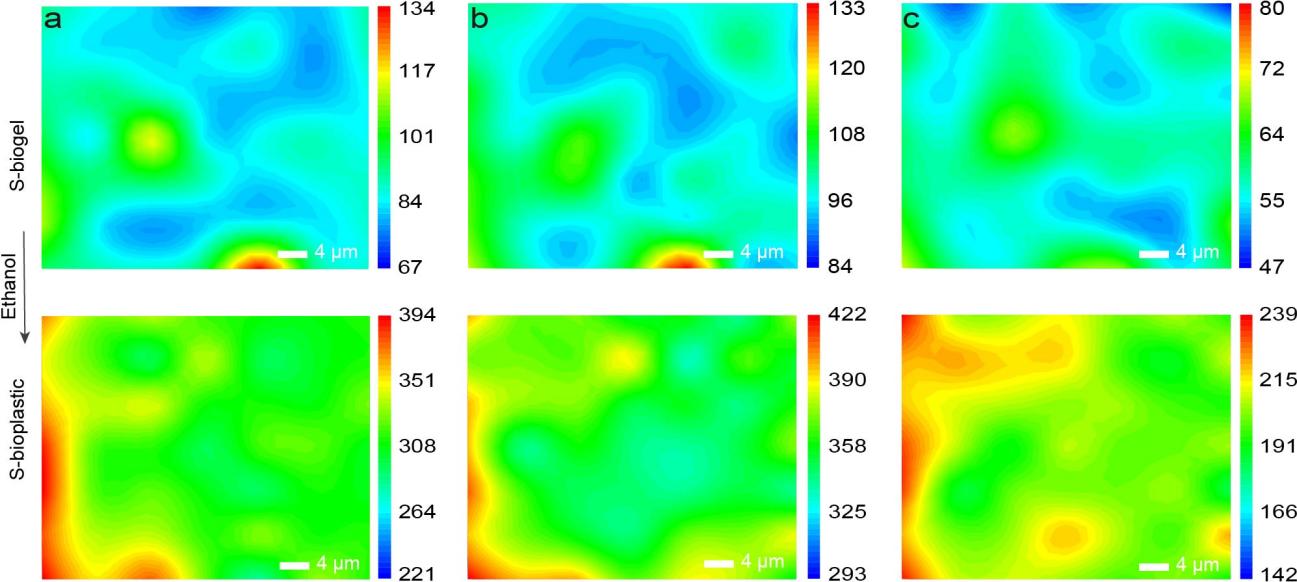
**

**Figure S8.** 2D Raman mapping of ring-internal C-O-C (a), glycosidic bond C-O-C (b), and

C-O (c).

2D Raman mapping confirmed the abundant presence of ring-internal C-O-C, glycosidic bond C-O-C, and C-O groups on S-bioplastic surfaces (high intensity indicated by the yellow and red), while S-biogel showed low-intensity (blue and green regions) 2D images.
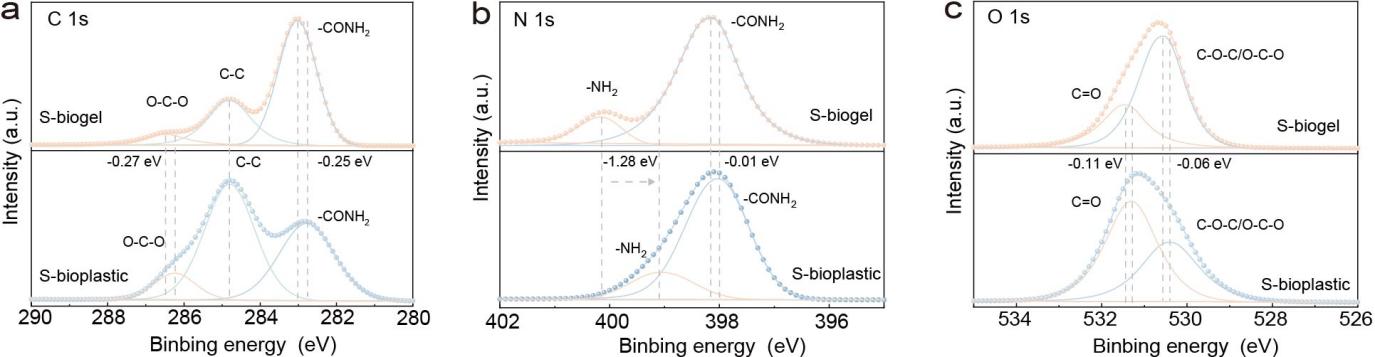


**Figure S9.** Comparation of the XPS spectra of C1s (a), N1s (b), and O1s (c) between the S-biogel and the S-bioplastic.

The XPS spectra showed that the Characteristic peaks of S-bioplastic all shifted to lower binding energy, which was caused by the gradual pulling out of water molecules from the S-biogel by ethanol. This process undoubtedly freed up -OH groups of cellulose and -NH_2_ groups of PAM, forming more H-bonds between cellulose and PAM.


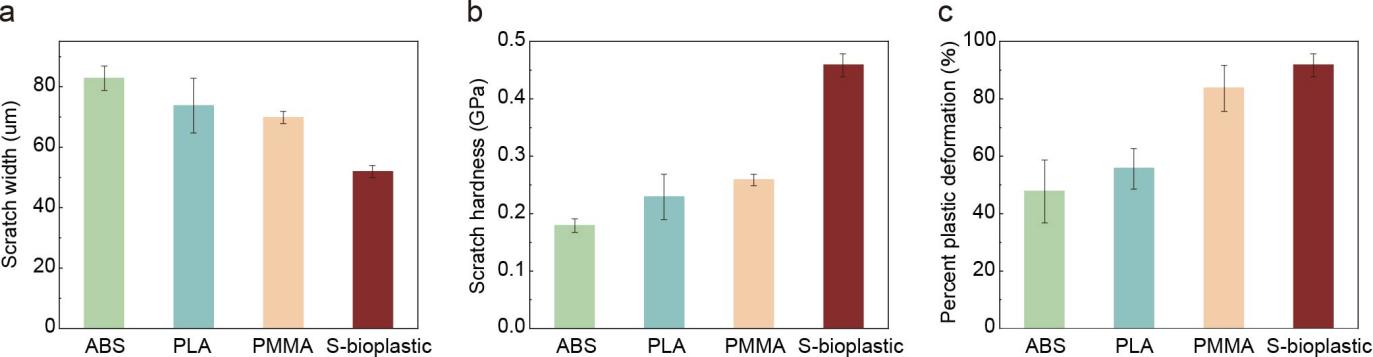


**Figure S10.** Scratch width (a), scratch hardness, and (b) plastic deformation rate (c) of the S-bioplastic after scratch tests, compared with commercial plastics.


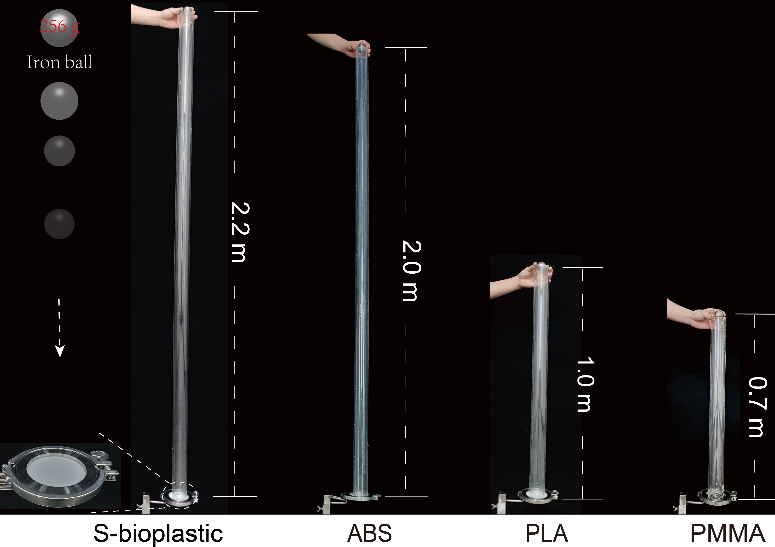


**Figure S11.** Optical photographs of free-fall impact performance testing for PMMA, PLA, ABS and S-bioplastic.


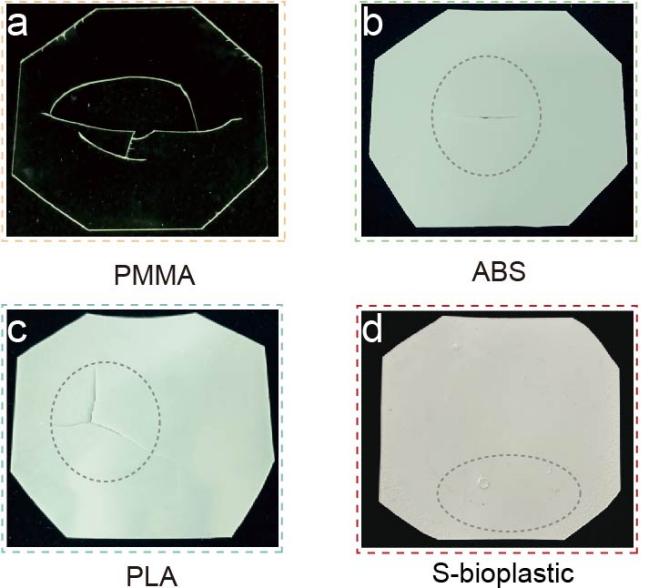


**Figure S12.** Photographs of PMMA, PLA, ABS, and S-bioplastic after the puncture tests.


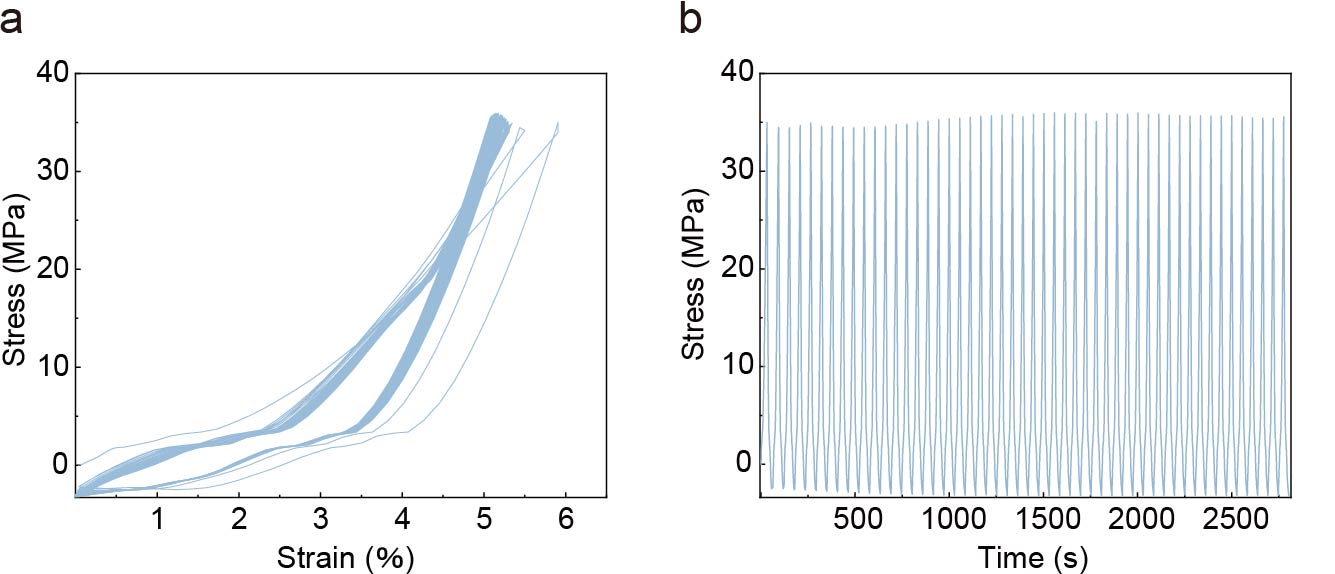


**Figure S13.** Tensile cycle curve of S-bioplastic. (a) Stress-strain cycle curve of S-bioplastic. (b) Stress-time cycle curve of S-bioplastic.

**
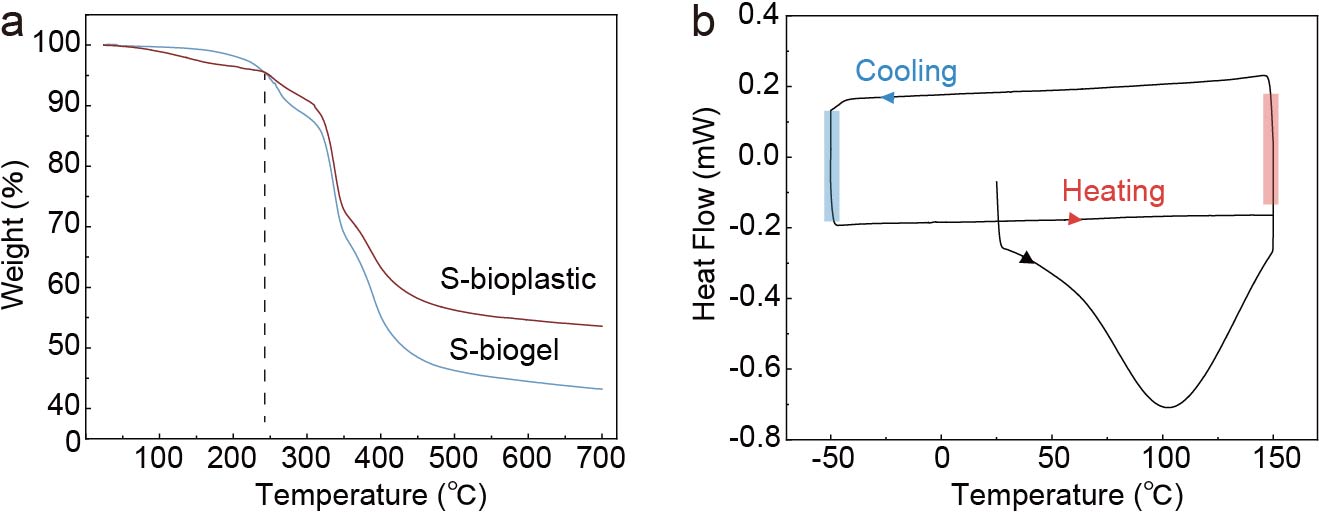
**

**Figure S14.** The thermal properties of S-bioplastic. (a) Thermogravimetric (TG) analysis of S-biogel and S-bioplastic. (b) Differential scanning calorimetry (DSC) curve of the S-bioplastic.


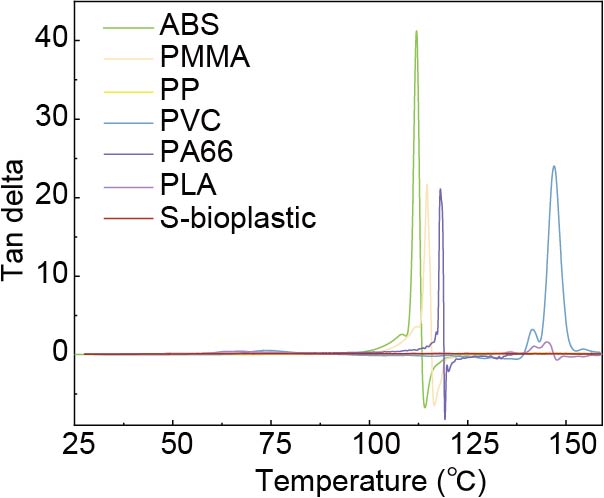


**Figure S15.** Investigating the thermal-mechanical properties of ABS, PMMA, PP, PVC, PA66, PLA, and S-bioplastic using thermomechanical analysis (DMA).


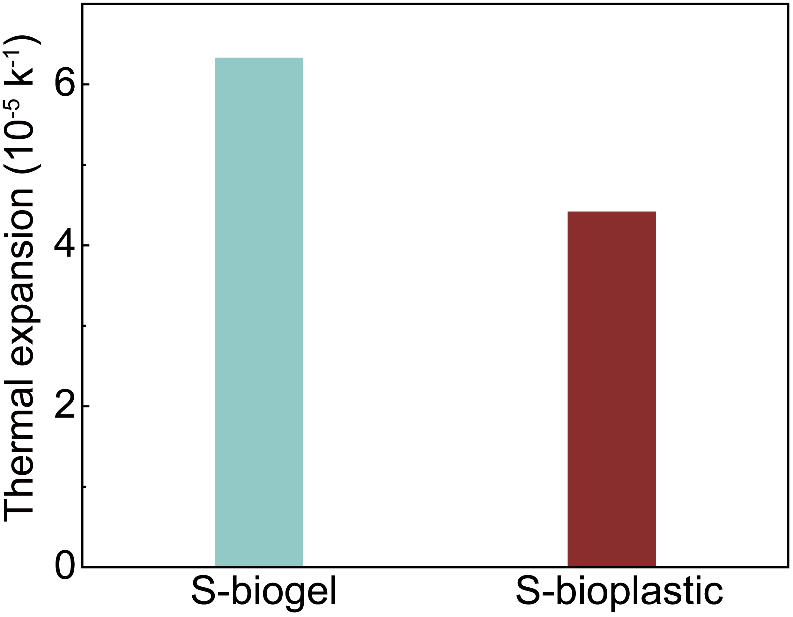


**Figure S16.** The thermal expansion coefficient of the S-biogel and S-bioplastic.


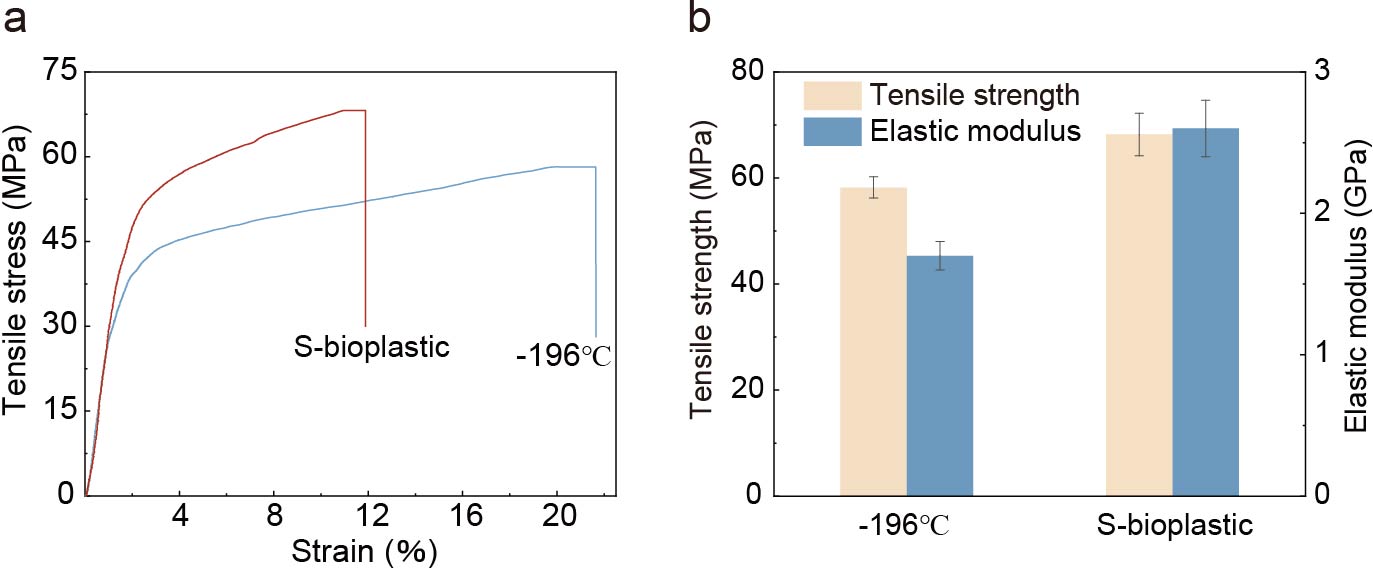


**Figure S17.** Tress-strain curves (a) and mechanical properties (b) of S-bioplastics before and after 120 s exposure to -196 °C liquid nitrogen.


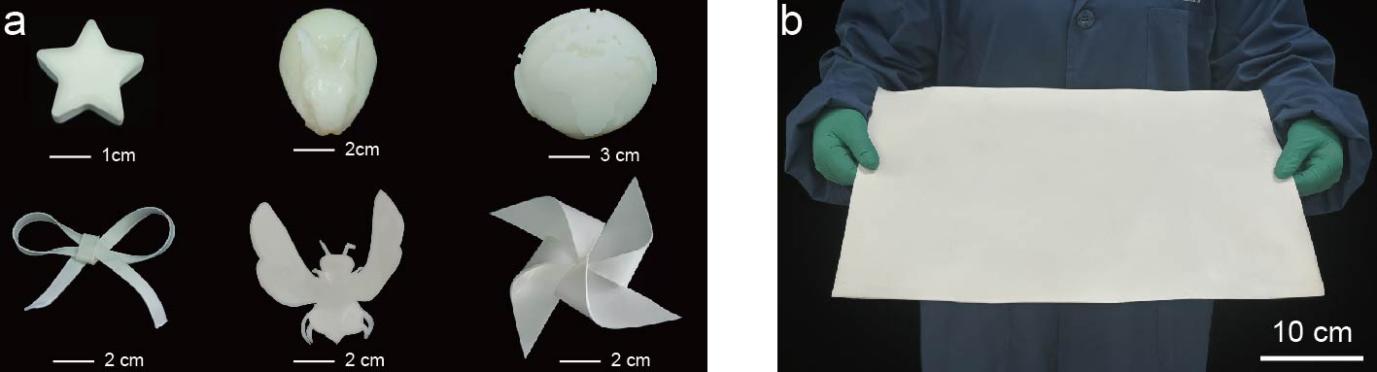


**Figure S18.** (a) Various molded S-bioplastic products. (b) Demonstration of the capability to process large-sized S-bioplastic.


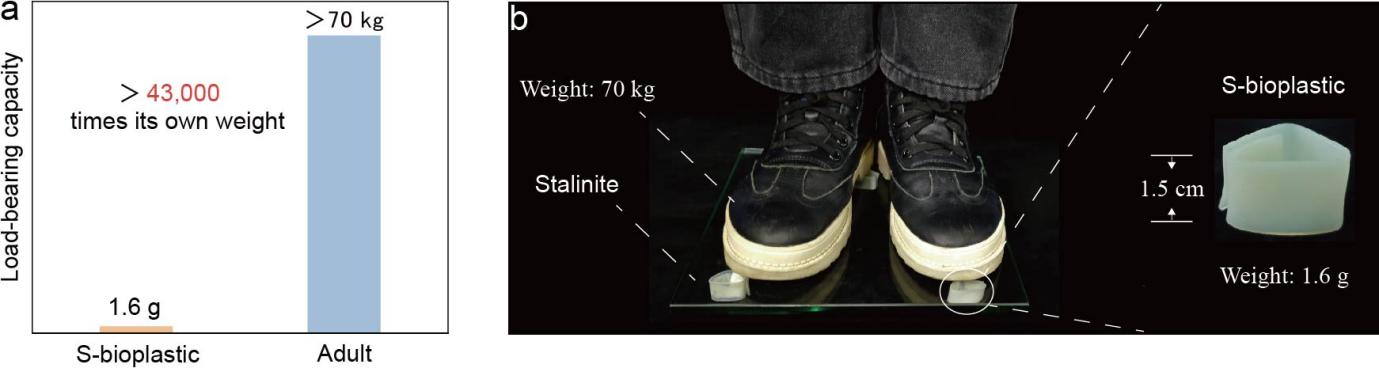


**Figure S19.** (a) S-bioplastic showing a perfect load-bearing capacity. (b) Optical images of the S-bioplastic, withstanding an over 70 kg weight.


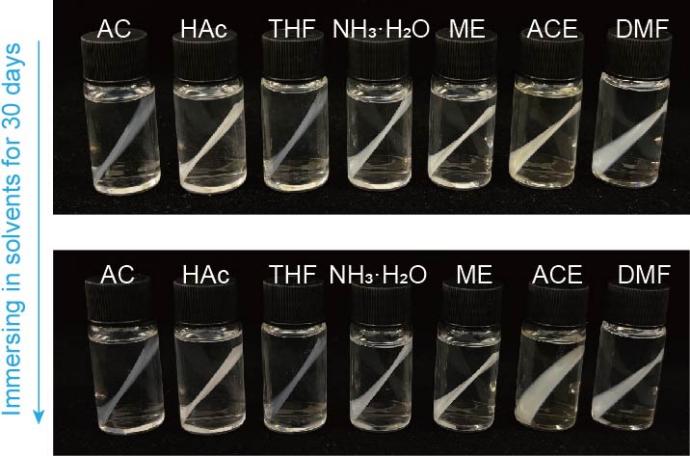


**Figure S20.** Digital images showcasing the shape retention of S-bioplastic after immersion in various solvents for 30 days.


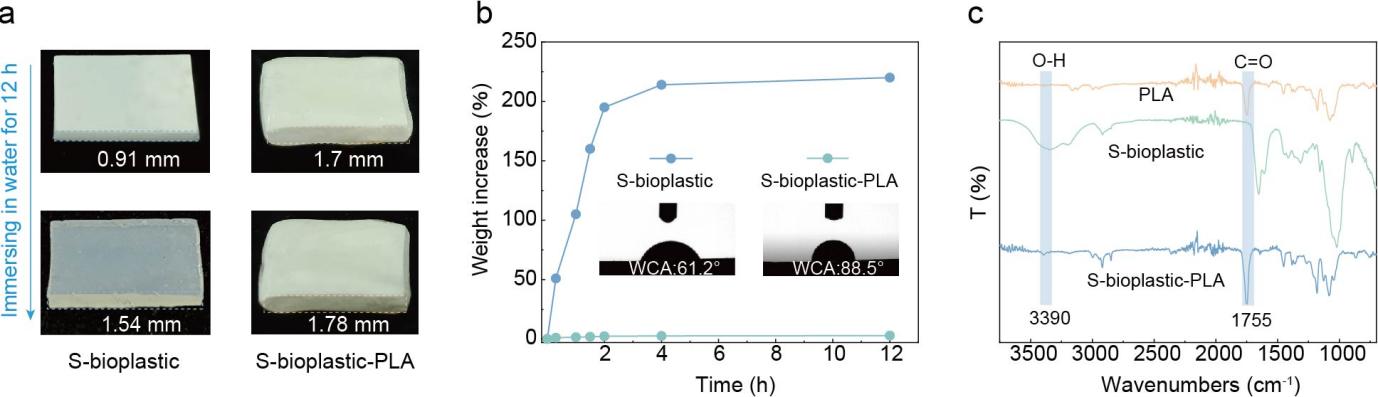


**Figure S21.** Surface characteristics of S-bioplastic before and after hydrophobic treatment by PLA. (a) Photographs of the comparison of S-bioplastic and S-bioplastic-PLA before and after 12 h water immersion. (b) Comparison of the weight increases of S-bioplastic and S-bioplastic-PLA during 12 h of water, the WCA showing a significant improvement in the hydrophobicity of S-bioplastic. (c) The FTIR spectroscopy of the S-bioplastic and S-bioplastic-PLA.


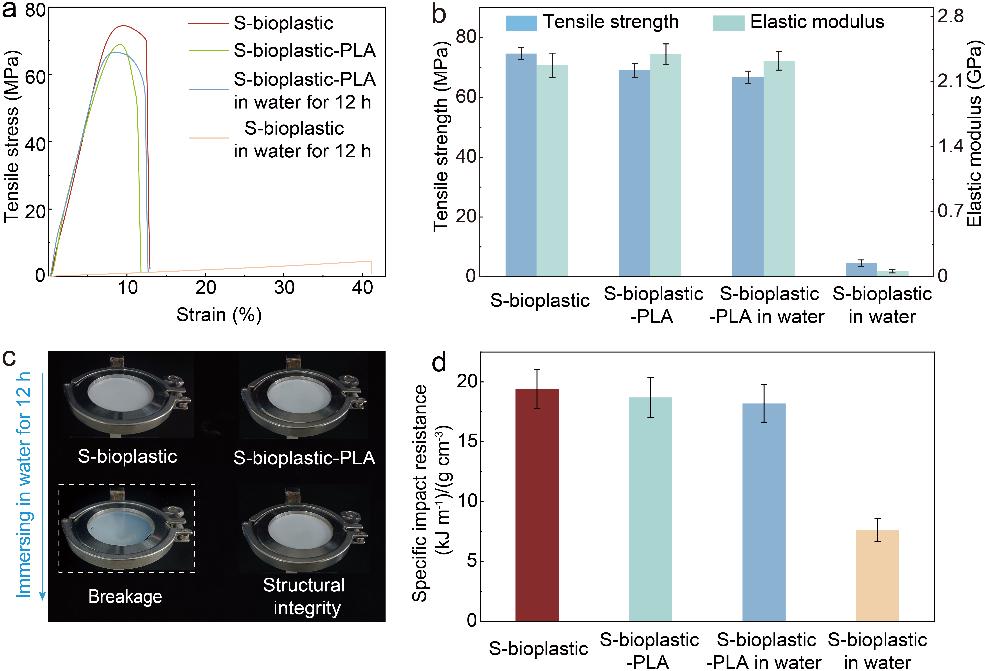


**Figure S22.** Mechanical properties of S-bioplastic and S-bioplastic-PLA before and after immersing. (a) Stress-strain curve. (b) Tensile strength and elastic modulus. (c) Digital photos of impact performance test. (d) Impact resistance.


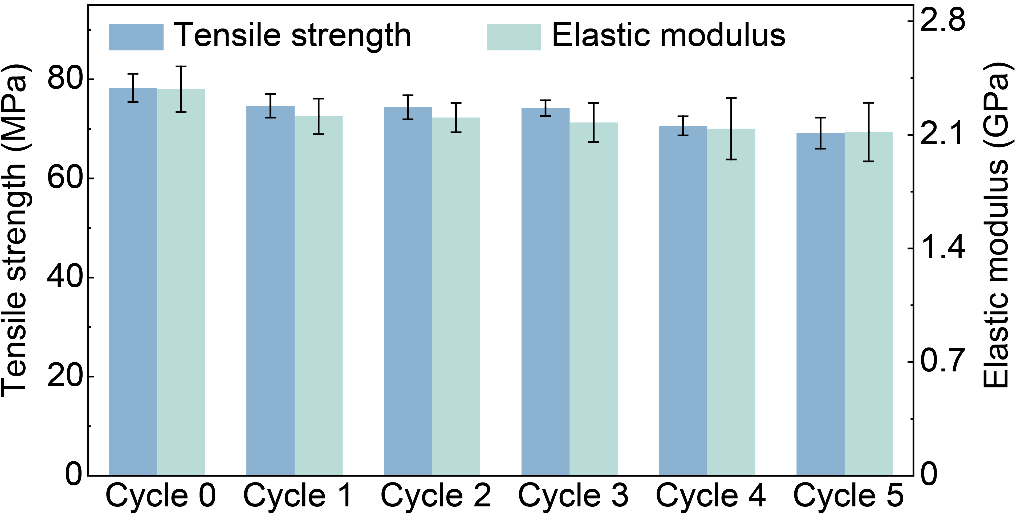


**Figure S23.** The tensile strength and elastic modulus of recycled S-bioplastic.

**Table S1.** Comparative analysis of mechanical properties of S-bioplastic after 5 cycles.

| **Cycle number** | **Tensile strength (MPa)** | **Elastic modulus (GPa)** |
| --- | --- | --- |
| Cycle 0 | 78.28 ± 2.83 | 2.381 ± 0.14 |
| Cycle 1 | 74.67 ± 2.39 | 2.214 ± 0.11 |
| Cycle 2 | 74.36 ± 2.43 | 2.206 ± 0.09 |
| Cycle 3 | 74.19 ± 1.58 | 2.176 ± 0.12 |
| Cycle 4 | 70.61 ± 1.94 | 2.138 ± 0.19 |
| Cycle 5 | 69.15 ± 3.12 | 2.117 ± 0.18 |

**Table S2.** Comparative prices among S-bioplastic, PMMA, ABS, PP, and PLA.

| **Materials** | **Price ($/t)** | **Source** |
| --- | --- | --- |
| PMMA | 2100 | https://www.alibaba.com/product-detail/High-Quality-Poly-Methyl-Methacrylate-Sheet_1600161027202.html?spm=a2700.galleryofferlist.normal_offer.d_title.513b13a0dM6ujY&priceId=21696ffde62a45d39acc2c1867ffee00 |
| ABS | 1500 | https://www.alibaba.com/product-detail/Resin-POLYLAC-PA757-PA-757-High_1600790871882.html?spm=a2700.galleryofferlist.p_offer.d_title.19d24bc4XmNMHB&s=p |
| PP | 1100 | https://www.alibaba.com/product-detail/Pp-Ep300h-PP-3090-PPK8009-PP_1601299875592.html?spm=a2700.galleryofferlist.p_offer.2.da2213a01028h3&priceId=d27739d0d73f4fc8821e5ba309b8cd7a |
| PLA | 2850 | https://www.alibaba.com/product-detail/ZOVGOV-100-BioPlastic-Compostable-PLA-Resin_1601028302759.html?spm=a2700 |
| S-bioplastic | 2987 | This work |

**Table S3.** Price estimation for S-bioplastic after 7 cycles. Each cycle is involving loss 17.4 wt% [Bmim]Cl, 20 wt% ethanol, and 20 wt% water.

| **Feedstocks** | **Price ($/t)** | **Consumption**  **(t)** | **Cost**  **($)** | **Source** |
| --- | --- | --- | --- | --- |
| Bamboo pulp | 800 | 0.56 | 448.05 | https://www.alibaba.com/product-detail/Natural-Bleaching-Bamboo-Paper-Pulp_60432515971.html?spm=a2700 |
| [Bmim]Cl | 2000 | 0.839 | 1678.89 | https://www.alibaba.com/product-detail/1-Butyl-3-methylimidazolium-Chloride-CAS_1601300481883.html?spm=a2700 |
| Water | 0.33 | 27.15 | 8.96 | https://scj.shenyang.gov.cn/zwgk/fdzdgknr/gsgg/202503/t20250331_4829895.html |
| PAM | 1000 | 0.3375 | 337.53 | https://www.alibaba.com/product-detail/Hot-Selling-Acrylamide-Manufacturers-AM-79061_1601407024941.html?spm=a2700 |
| Ethanol | 800 | 0.504 | 403.25 | https://chinese.alibaba.com/product-detail/Ethanol-Alcohol-Perfume-Used-High-Quality-10000014052937.html?spm=a27aq |
| Electricity | 0.07 | 1578.85 | 110.52 | https://scj.shenyang.gov.cn/zwgk/fdzdgknr/gsgg/202503/t20250331_4829895.html |
| Total cost | 2987 | | | |
